# Hepatic histomorphological and biochemical changes following highly active antiretroviral therapy in an experimental animal model: Does *Hypoxis hemerocallidea* exacerbate hepatic injury?

**DOI:** 10.1016/j.toxrep.2015.12.013

**Published:** 2016-01-06

**Authors:** Onyemaechi Okpara Azu, Ayoola Isaac Jegede, Offor Ugochukwu, Ismail Olasile Onanuga, Salem Kharwa, Edwin Coleridge Naidu

**Affiliations:** a Discipline of Clinical Anatomy, School of Laboratory Medicine and Medical Sciences. Nelson R Mandela School of Medicine, University of KwaZulu-Natal, Durban 4001, South Africa; b Anatomy Department, Faculty of Basic Medical Sciences, College of Health Sciences, Ladoke Akintola University of Technology, Ogbomoso, Osun, Nigeria; c Department of Anatomy, Faculty of Basic Medical Sciences, Kampala International University, Kansaga, Ggaba Road, Uganda

**Keywords:** Liver morphology, HAART, Cytotoxicity, Stains, Biochemistry, Lipid profile

## Abstract

As the roll-out of antiretroviral therapy continues to drive downwards morbidity and mortality in people living with HIV/AIDS (PLWHAs), organ toxicities (especially the liver) are frequently becoming a major concern for researchers, scientists and healthcare planners.

This study was conducted to investigate the possible protective effect of *Hypoxis hemerocallidea* (AP) against highly active antiretroviral therapy (HAART)-induced hepatotoxicity. A total of 63 pathogen-free adult male Sprague-Dawley rats were divided into 9 groups and treated according to protocols.

While no mortality was reported, animals treated with adjuvant HAART and AP recorded least% body weight gain. Significant derangements in serum lipid profiles were exacerbated by treatment of with AP as LDL (increased *p* < 0.03), triglycerides (increased *p* < 0.03) with no change in total cholesterol levels. Adjuvant AP with HAART caused reduction in LDL (*p* < 0.05 and 0.03), increased HDL (*p* < 0.05) and TG (*p* < 0.05 and 0.001 for AP_100_ and AP_200_ doses respectively). Markers of liver injury assayed showed significant increase (*p* < 0.003, 0.001) in AST in AP alone as well as HAART+ vitamins C and E groups respectively. Adjuvant HAART and AP and vitamins C and E also caused significant declines in ALT and ALP levels. Serum GGT was not markedly altered. Disturbances in histopathology ranged from severe hepatocellular distortions, necrosis and massive fibrosis following co-treatment of HAART with vitamins C and E as well as HAART alone. These results warrant caution on the adjuvant use of AP with HAART by PLWHAs as implications for hepatocellular injuries are suspect with untoward cardiometabolic changes.

## 1 Introduction

The acquired immuno-deficiency syndrome (AIDS) is a significant threat to the health of mankind and the search for effective therapies to treat AIDS is of paramount importance. The development and evolution of therapy against the human immune deficiency virus (HIV) therapy (which causes AIDS), has tremendously improved over the last 2 decades with resultant significant increase in life expectancy among HIV-infected patients [1], [2]. Highly active antiretroviral therapy (HAART), a combination of this chemotherapeutic regime for people living with HIV/AIDS (PLWHAs), suppresses viral replication but its major drawback is adverse effects of toxicities to organs [2].

Hepatotoxicity has been associated with most antiretroviral drugs like Zidovudine, Stavudine or Didanosine [3] and potentially contributes in part to poor compliance and adherence necessitating switch with therapy [2]. The liver performs the function of formation of excretory products, degradation of complex cellular materials, and the synthesis of plasma proteins. Due to this functional complexity, any change in liver function and histoarchitecture becomes an important consideration for study of abnormality [4].

Besides the high cost of HAART regimens, the adverse effects associated with using chemotherapy for the treatment of HIV infection have further encouraged the utilization of herbal medicines as an alternative medical therapy by PLWHAs [5]. This complementary or alternative therapy is thought to mitigate this toxic effect of HAART regimens.

*Hypoxis hemerocallidea* commonly known as African potato (AP), also known as *Hypoxis rooperi* (Hypoxidaceae), has a long history of traditional use for a diversity of ailments [6] and more recently has been the subject of several scientific studies. In many parts of Africa, the corms of this attractive yellow flowered herb have been used in the treatment of urinary diseases, prostate hypertrophy and cancer [7] and more recently as immune boosters for HIV–AIDS. Its traditional usage dates back many generations [8] and anecdotal evidence indicates that the plant can be poisonous [9]. The AP is noted for the occurrence of a hypoxoside, which is a secondary metabolite of the plant [7] that is hydrolyzed into rooperol in vivo—the active and powerful antioxidant component of the corm [10] in the large intestine.

Pharmacokinetic studies have indicated that rooperol can be found in feces, and metabolites are found in the serum and urine as its glycosides, sulfates, mixed glucuronides, and sulfuronides [11]. These metabolites, when conjugated back to rooperol, were found to be cytotoxic to cancerous cells [7]. The glycoside has low toxicity and the corm containing it is also used as food [6] and has been well used for traditional and pharmaceutical purposes [8].

With increasing interest in the use of phytosterols (one of phytochemical components of AP) for the reduction of serum cholesterol and for immune boosting, there has been a resultant increase in scientific investigations [12] surrounding these benefits. Interestingly, there has been a surge in commercially available herbal medicines containing sterols with AP extract enrichments claimed to be efficacious against a variety of diseases. However, the scientific validation of these claims remains to be verified despite its anti-inflammatory, antimicrobial, antidiabetic, anticonvulsant and anticancer properties reported by various authors [13], [14], [15] but none on any antiretroviral-based therapy. As a result, there is paucity of literature explaining its attenuating influence on liver associated with HAART.

Therefore, we investigate the role of crude aqueous extracts of *H. hemerocallidea* (AP) on the histoarchitecture of the liver, glycogen distribution, degree of fibrosis and hepatocellular functional indexes of animal experimental protocol following HAART.

## 2 Materials and methods

### 2.1 Chemicals/drugs

Lamivudine (3TC), Stavudine and Nevirapine (Aspen) and vitamin C (L-ascorbic acid) were procured from Pharmacare Ltd, Port Elizabeth, South Africa and are of analytical grade. Vitamin E solution was obtained from Kyron Prescription CC, Benrose in Johannesburg.

### 2.2 Plant

Fresh corms of AP were purchased from a local ‘Muthi’ shop in Umbilo Road, Durban, KwaZulu-Natal, between June and July, 2014. The corms were authenticated at the Department of Life Science, Westville Campus, University of KwaZulu-Natal, South Africa.

### 2.3 Preparation of corm aqueous extract

AP fresh corms were extracted according to the procedure of Ojewole et al. [16]. They were washed with water, cut into smaller pieces, air dried at room temperature (25–28 °C) and ground into powdered form in a commercial blender. The milled corm (250 g) was soaked in hot distilled water and extracted twice, on each occasion with 2.5 L of hot distilled water (at 90–100 °C) for 12 h. The combined extract soluble were concentrated to dryness under reduced pressure in a rotary evaporator at 70 ± 1 °C. The resulting crude aqueous extract was freeze dried, finally giving of a dark brown, and powdery aqueous extract residue. Without any further purification, aliquot portions of the aqueous extract residue were weighed and dissolved in distilled water (at room temperature) for use on each day of our experiments.

### 2.4 Ethics approval, animal treatment and experimental design

Sixty three pathogen-free adult male Sprague-Dawley rats (aged 9–10 weeks old) and weighing between 256 and 312 g were used for this study. The animals were bred and maintained at the Animal House of the Biomedical Resources Unit, University of KwaZulu-Natal. The animals received humane care in accordance with the Principle of Laboratory Animal Care of the National Medical Research Council and the Guide for the Care and Use of Laboratory Animals of the National Academy of Sciences (National Institute of Health Guide, 1985). The study protocol was approved by the University of KwaZulu Natal Animal Ethics Committee (Ethics clearance number: 100/14/Animals). All the rats were housed in well ventilated plastic cages (2 rats per cage) having dimensions of (30 cm long × 20 cm wide and 13 cm high). They were maintained under standardized animal house conditions (temperature: 21–23 °C; light: approximately 12 h light per day) and were fed with standard rat pellets (from Meadow feeds a Division of Astral Operations Limited, Durban, South Africa) and given tap water *ad libitum*. The animals were randomly distributed to nine treatment groups; A, B, C, D, E, F, G, H and I and treated as indicated below.

Group A: HAART (a cocktail of Lamivudine, Stavudine and Nevirapine) using recommended human therapeutic doses and accordingly adjusted to the equivalent animal dose (stavudine 0.57, lamivudine 2.06 and nevirapine 1.54 mg/kg body weight respectively).

Group B: received HAART and AP (100 mg/kg body weight)

Group C received HAART and AP (200 mg/kg body weight)

Group D received HAART and Vitamin C (250 mg/kg body weight)

Group E received HAART and Vitamin E 40 mg/kg body weight [17]

Group F: Combination of HAART, Vitamins C and E.

Groups G and H received AP extract alone at doses of 100 and 200 mg/kg respectively.

Groups I served as the control administered 0.9% normal saline.

All administration was done daily by oro-gastric gavage except for vitamin E which was administered subcutaneously. At the end of the treatment period (56 days), the animals were killed 24 hours after the last treatment under excess Halothane^®^ anesthesia.

### 2.5 Body and liver weight

Body weights of animals were recorded on the first day before treatment (initial), thereafter weekly and on the day of sacrifice (final). Liver weight (LW) was measured by an electronic balance (Mettler Toledo; Microsep (Pty) Ltd, Greifensee, Switzerland).

### 2.6 Assessment of liver function and lipid profile

Blood samples were collected through cardiac puncture and allowed to cloth for 30 min and centrifuged for 15 min at 3000 revolutions per minute. The serum was decanted into Eppendorf tubes and prepared for biochemical analyses.

Biochemical analyses of the serum enzymes for alanine aminotransferase (ALT), aspartate aminotransferase (AST), alkaline phosphatase (ALP) and gamma-glutamyl transferase (GGT) were spectrophotometrically determined by the method of Reitman and Frankel [18].

### 2.7 Determination of serum lipids

Low density lipoprotein (LDL), high density lipoprotein (HDL), Triglycerides (TG) and total cholesterol (CHOL) were determined by enzymatic methods according to Diniz et al. [19] using commercial diagnostic kits (Randox, UK).

### 2.8 Histopathological examination of liver tissues

Twenty four hours after the last treatment the animals were euthanized under excess Halothane^®^ anesthesia and the liver tissues were excised, blotted of blood and weighed. They were examined for gross pathology and immediately fixed in 10% Neutral buffered formalin. After proper fixation, the tissues were dehydrated in graded series of alcohol, cleared in Xylene and embedded in paraffin wax using a cassette.

For routine histological study, the liver tissues were sectioned at 5 μm thickness using Leica RM 2255 microtome and stained with hematoxylin and eosin (H&E) for general assessment of liver structure. For histochemical studies, the tissues were stained with Periodic acid Schiff’ (PAS) for glycogen, neutral polysaccharides and basement membrane, and Masson trichrome (MT) for the assessment of possible liver fibrous architecture [20]. The stained slides were then cover slipped using DPX mounting glue directly over the tissue section ensuring no air bubbles were trapped. Thereafter, the slides were left overnight to dry for examination under light microscope.

The sections were examined using a binocular microscope and image acquired using the Nikon Eclipse 80i (Tokyo, Japan). An independent Histopathologist blinded to the treatment groups reported on the qualitative assessments of the slides.

### 2.9 Statistical analysis

Continuous variables (liver and body weights, liver function test and lipid profile level), were analyzed by one-way analysis of variance (ANOVA) followed by Dunnett’s multiple comparison post-test using Graph pad prism^®^ statistical software 6.02. The results are expressed as mean ± SD (standard deviation). Values were considered significant at *p* < 0.05.

## 3 Results

### 3.1 Body weight and organ (liver) weight changes

While the final body weight of animals in all groups were higher than their corresponding initial body weight, the percentage weight gain was maximal in group D (HAART with vitamin C), then groups H and G all recording 48.66%, 46.38% and 44.08% respectively. Least percentage weight gain was observed in animals treated with HAART + AP 100 mg/kg (29.89%) (Table 1). There was a significant decline in final body weight in groups C and F (*p* < 0.05), G and H (*p* < 0.03) when compared with control. There were no significant (*p* > 0.05) increase in liver weight in group A compared with the control. Group F recorded the lowest liver weight when compared with the control (*p* < 0.03). Similarly, organ-body weight ratios were significantly decreased in groups B, C, E (*p* < 0.05), D and F (*p* < 0.03) (Table 1).

**Table 1:** Body and liver weight changes in experimental groups.

Group	Treatment	Initial (g)	Final (g)	BW diff (g)	% BW diff	LW (g)	LBWR
A	HAART	285.86 ± 3.80	407.29 ± 13.69	115·57	37·11	14.18 ± 3.41	0.035
B	HAART + AP_1_	311.43 ± 3.56	427.00 ± 09.94	83·57	29·89	12.05 ± 1.49	0.028*
C	HAART + AP_2_	279.57± .16	363.14 ± 11.50**	101·14	35·92	10.05 ± 0.98*	0.028*
D	HAART + vit C	281.57 ± 2.88	382.71 ± 05.51	127·00	48·66	10.08 ± 0.76*	0.026**
E	HAART + vit E	261.00 ± 7.49	388.00 ± 08.28	88·14	32·46	10.66 ± 0.72*	0.027*
F	HAART + C + E	271.57 ± 7.14	359.71 ± 12.31**	98·86	36·21	09.41 ± 1.27**	0.026**
G	AP_1_	273.00 ± 6.29	371.86 ± 18.43*	113·15	44·08	13.16 ± 2.38	0.035
H	AP_2_	256.71 ± 8.65	369.86 ± 12.66*	121·00	46·38	12.83 ± 1.31	0.035
I	Control	260.00 ± 6.06	381.00 ± 11.21	121·43	42·48	13.78 ± 1.06	0.036

*p < 0.05; **p < 0.03 compared with control; BW = body weight of rats, LW = liver weight of rats, LBWR = liver-body weight ratio; AP_1_/AP_2_-100 and 200 mg/kg dosage.

### 3.2 Serum AST, ALT, ALP and GGT in the experimental study

There were changes in the functional hepatotoxicity indices recorded via AST, ALT ALP and GGT. AST levels were reduced in groups A and E (not significant) when compared with the control, it was significantly elevated in groups F and H (HAART with vitamin C&E/AP 200 mg/kg) respectively (*p* < 0.001, 0.03) compared with the control. There was statistically significant decrease in ALT levels in groups A–E (*p* < 0.03) whereas the levels in groups F, G and H were lower than that of control (*p* > 0.05). Similarly, ALP levels of animals treated with HAART, HAART + AP (both doses), and HAART + vitamin E (*p* < 0.001), HAART + vitamin C, AP (both does) (*p* < 0.03) were significantly lower compared with the control. The results of the GGT levels were not statistically significant in all groups when compared with the control group (*p* > 0.05) (Table 2).

**Table 2:** Effect on serum AST, ALT ALP and GGT following treatment with HAART and AP.

Group	AST (IU/L)	ALT (IU/L)	AST/ALT ratio	ALP (IU/L)	GGT (IU/L)
A	92.00 ± 3.23	48.67 ± 11.91***	1.89	116.67 ± 41.55***	2.00 ± 0.89
B	106.33 ± 7.45	52.00 ± 2.37***	2.04	95.00 ± 5.59***	3.00 ± 0.00
C	108.00 ± 9.30	51.33 ± 8.96***	2.10	88.00 ± 4.98***	3.00 ± 0.89
D	104.67 ± 4.03	55.33 ± 7.61***	1.89	122.67 ±16.79**	3.33 ± 0.52
E	94.33 ± 13.55	44.00 ± 3.10***	2.14	99.67 ± 4.03***	3.00 ± 0.89
F	128.33 ± 6.83***	75.67 ± 17.6	1.70	167.00 ± 5.87	2.67 ± 0.52
G	105.33 ± 11.67	75.00 ± 8.53	1.40	160.67 ± 36.88*	3.00 ± 0.89
H	116.00 ± 13.00**	75.67 ± 8.31	1.53	145.33 ± 23.62**	2.67 ± 1.37
I	96.33 ± 9.48	79.67 ± 9.97	1.21	218.00 ± 71.52	2.33 ± 0.52

*p < 0.05, **p < 0.03, ***p < 0.001 compared with control, AST = alanineamino aspartate, ALT = alanine aminotransferase, ALP = alkalinephosphatase, GGT = gamma-glutamyl transferase.

### 3.3 Effect on lipid profile following treatment with HAART and AP

Although total cholesterol levels in all groups were slightly higher than control values, these were not significantly different (*p* > 0.05). There were statistically significant increase in the serum levels of LDL in groups F and H (*p* < 0.03), whereas groups treated with HAART, HAART + vitamin C (*p* < 0.001), HAART + AP100 mg/kg, HAART + vitamin E (*p* < 0.05) as well as HAART + AP200 mg/kg (*p* < 0.03) all recorded significant decline in the parameter compared with the controls. HDL levels in groups A, C, E (*p* < 0.05) as well as in groups D, F and H (*p* < 0.03) were all significantly lower than the control group. While triglyceride (TG) levels in groups A, C, D, E (*p* < 0.001), B (*p* < 0.05) statistically declined, levels were however increased in the groups administered HAART + vitamins C and E (*p* < 0.001) as well as AP200 mg/kg (*p* < 0.03) (Table 3).

**Table 3:** Lipid profile in experimental groups.

Group	LDL (mmol/L)	HDL (mmol/L)	CHOL (mmol/L)	TG (mmol/L)
A	−0.10 ± 0.02***	1.00 ± 0.05*	1.10 ± 0.09	0.45 ± 0.09***
B	−0.23 ± 0.13*	0.94 ± 0.06	1.07 ± 0.14	0.68 ± 0.16*
C	−0.12 ± 0.09**	0.98 ± 0.03*	1.10 ± 0.09	0.52 ± 0.13***
D	−0.09 ± 0.06***	1.04 ± 0.11**	1.17 ± 0.14	0.45 ± 0.14***
E	−0.17 ± 0.03*	0.97 ± 0.05*	1.03 ± 0.05	0.51 ± 0.04***
F	−0.62 ± 0.01**	1.06 ± 0.05**	1.13 ± 0.05	1.50 ± 0.04***
G	−0.27 ± 0.03	0.92 ± 0.05	1.07 ± 0.05	0.90 ± 0.01
H	−0.63 ± 0.13**	1.06 ± 0.06**	1.07 ± 0.05	1.39 ± 0.30**
I	−0.38 ± 0.17	0.67 ± 0.47	1.03 ± 0.14	1.03 ± 0.23

*p < 0.05, **p < 0.03, ***p < 0.001 compared with control, LDL = low density lipoprotein, HDL = high density lipoprotein, CHOL = total cholesterol, TG = triglycerides.

### 3.4 **Histopathology of liver sections (H&E, Masson**’**trichrome and PAS techniques)**

**Fig. 1:**
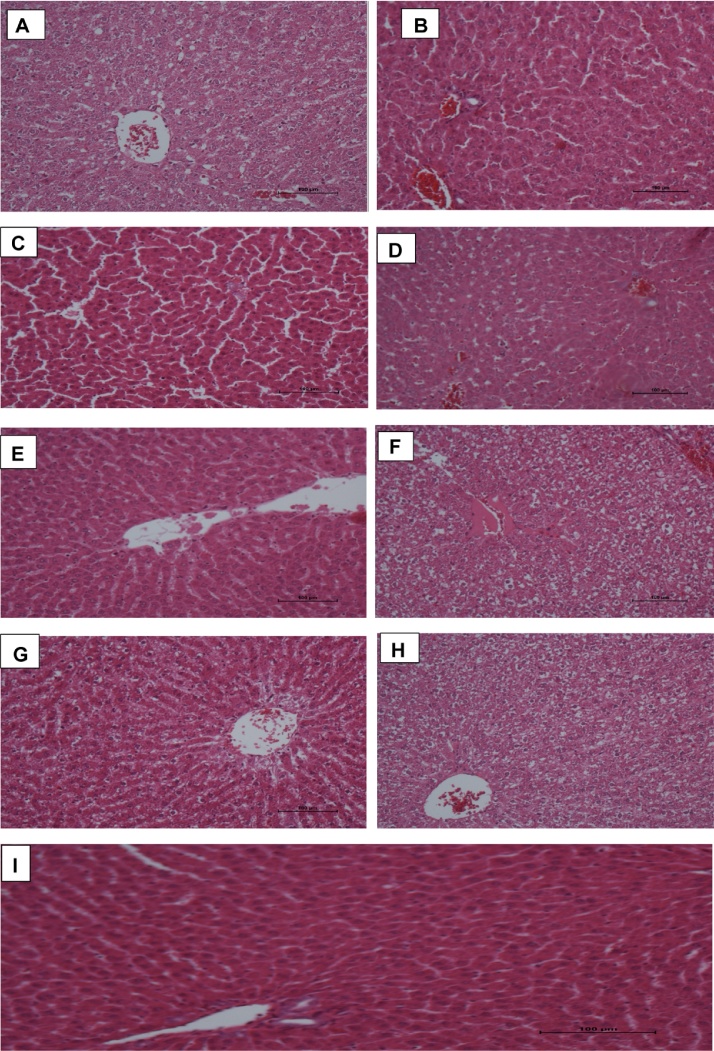
(A–I) Photomicrographs of liver sections stained with H & E. Note the normal architecture of hepatocellular cords sinusoidal spaces and central vein in groups E and I. There are various degrees of distortion (mild to severe) in the radial arrangement of hepatic cords seen in groups B–D. extensive necrosis are observed in groups F, H, A and G.

In H&E stained slides, control sections of liver showed typical hepatocellular architecture with central vein (containing blood) and cords of hepatocytes arranged peripherally in a radiating fashion. The outlines of hepatocytes and sinusoidal spaces are clearly seen with no obvious pathologies. This trend was observed in liver sections from groups E (HAART + vitamin E). Histopathological assessment of other groups revealed various degree of distortions. Liver tissues of group B animals showed mild distortion in the radial arrangements of hepatocytes and sinusoids with some form of ballooning of hepatocytes. Whereas in group C rats, some of the central veins appeared eroded with gradual loss of polyhedral arrangement of cords of cells. There was extensive necrosis of hepatocytes and sinusoidal spaces with marked loss of architecture in group D sections of liver. Some of the hepatocyte nuclei appeared enlarged. In sections of liver of group F animals, there was complete loss of architecture with hepatocytes appearing as isolated cell with (or without) prominent nucleoli. Groups treated with AP alone (both doses) showed liver sections that generally appeared enlarged in sinusoidal appearance with necrotic distortions (Fig. 1).

**Fig. 2:**
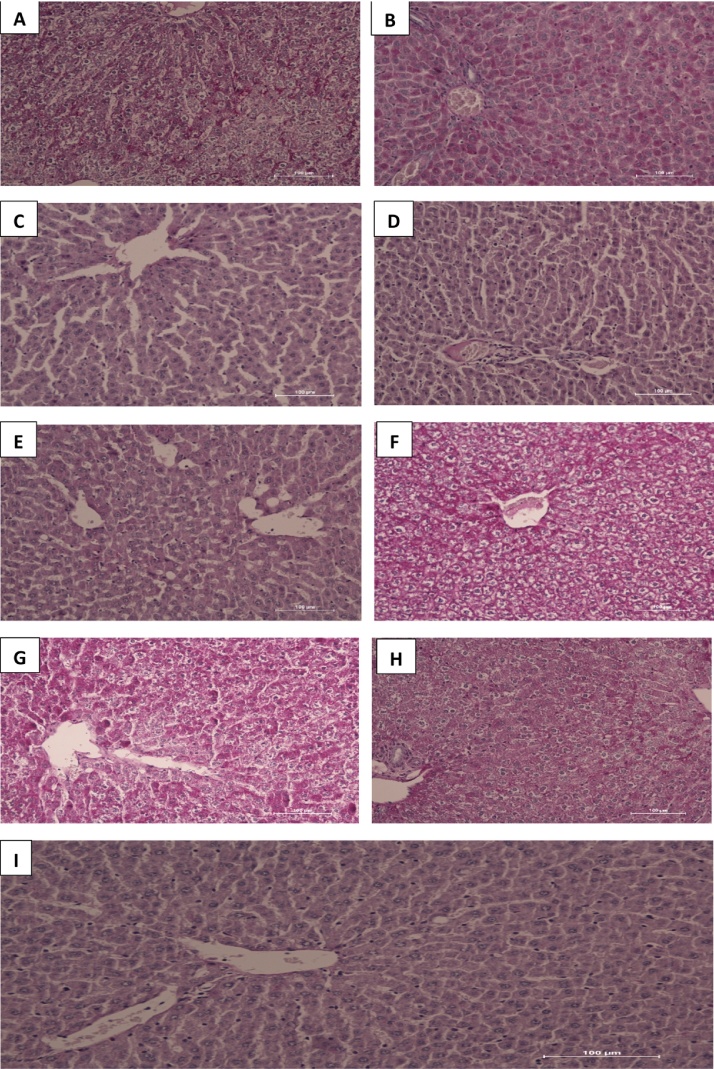
(A–I) Photomicrographs of liver sections stained with PAS. Note the intense pink colour and intensity of hepatocytes in groups F, G, D, A, H, E, B and C in decreasing order. There is hepatocellular cord derangements seen mostly in groups A, F, G and H.

PAS-stained sections reveal glycogen presence in the tissues and is depicted by pink color in the sections. Liver tissues from groups F, G, D, A, H, E, B and C showed decreasing PAS-staining intensity respectively with HAART + vitamins C and E treated groups having the maximum staining whereas HAART + AP200 mg/kg the least compared with controls. The extensive and generalized staining in slides F, G, D revealed large amount of glycogen in hepatocytes as well as in cords/sinusoidal spaces with bright pink hepatocytes, neutral polysaccharides and basement membranes completely PAS-positive in the sections. While the PAS stain demonstrates glycogen, indications for distortion in hepatocellular cords were evident in the grading of the intensity as well (Fig. 2).

**Fig. 3:**
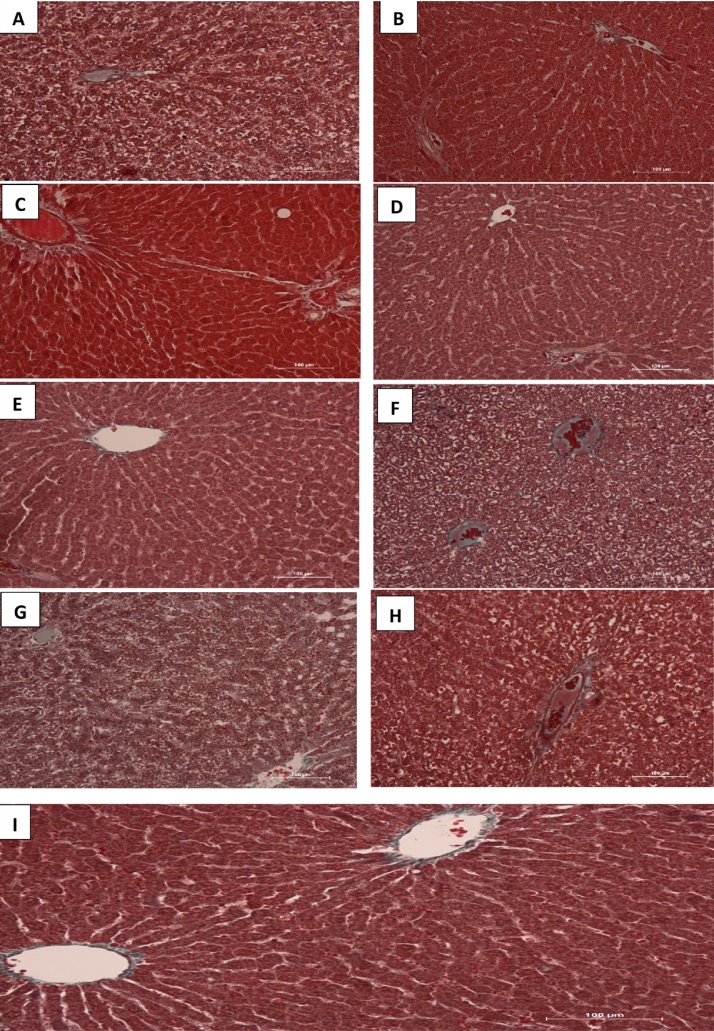
(A–I) Photomicrograph of the liver sections stained with Masson Trichrome (MT). Note the normal architecture of the liver with hepatocytes stained red, nuclei can be seen as dark red to black structures within cells and fibrous elements stained light blue in colour in the control group. There is extensive necrosis of hepatic cords and massive fibrosis in groups A, F, G and H.

Masson’s trichrome stains fibrous tissue blue while the cytoplasm of hepatocytes are stained red and nuclei could be dark or red as well. Sections of liver in control group showed normal red-stained hepatocytes and fibrous elements that are lightly stained blue especially around the central vein. Photomicrographs from liver tissues of groups treated with HAART + vitamins C + E as well as HAART alone showed extensive network of blue-stained fibrous components in a necrotized, disorganized hepatic tissue cord. In groups B–E, there were fine threads of blue-stained central veins and thin fibers across the cords. In many tissue sections, the central veins were well highlighted in their fibrous content and hepatocytes deeply stained (Fig. 3).

## 4 Discussion

As the roll-out of antiretroviral therapy continues to drive downwards morbidity and mortality in PLWHAs, organ toxicities (especially the liver) are frequently becoming a major concern for researchers, scientist and healthcare providers. Our current study revealed that the administration of HAART and *H. hemerocallidea* (AP) showed various perturbations in histological and biochemical indices which brings to attention the potential pitfalls in this adjuvant therapy. Due to the livers’ central role in the clearance and transformation of chemicals it is exposed to toxic injuries [21], [22]. In this study, organ-body weight ratio offers insight on toxicity/or inflammation [23] and whilst there were significant liver-body weight ratios especially in HAART with adjuvant AP/vitamins groups, these reductions may be attributed to cellular morphological changes and tissue movements [24]. Hepatomegaly has been noted with HAART, drawing caution on possible implications with individuals with co-existing liver pathologies.

Repeated oral administration of HAART and AP caused an increase in body weight of experimental animals mostly marked in HAART+ vitamin C group. While weight gain may be associated with the initiation of HAART possibly due to other metabolic issues related to lipid/sugar, the long term consequence is eventual weight loss [25]. Our results suggests that extracts of AP slowed the weight-gaining potential of the antiretroviral regimen as depicted in the results. The overall implication of this remains to be interpreted in the context of other results assessed. However, it is known that nucleotide reverse transcriptase inhibitors e.g., AZT (used in the cocktail) decreases the activity of cytochrome c oxidase and fatty acid synthase with consequent reduction in cellular lipid accumulation [26].

We assayed serum alanine aminotransferase (ALT) and aspartate aminotransferase (AST), alkaline phosphatase (ALP) and gamma-glutamyl transferase (GGT) in this protocol. Serum ALT and AST are helpful in understanding inflammatory and necrotic changes in the liver [27]. The increased levels of AST (especially following adjuvant HAART with AP/vitamins C and E) in our work supports previous reports by Oviosu et al. [25] on effects of AZT and nevirapine on liver enzymes. While the levels of ALT were reduced in our study, AST levels were on the higher level. Elevation in the level of amino transferases are associated with the commencement of antiretroviral therapy in humans [28] and can be exacerbated by other co-infections like hepatitis B or C virus [28], [29], [30]. The high concentration of both amino transferases implies that increased activities reflects a major permeability problem or cell disruptions in the liver [31].

In the quest for better outcome and well-being of PLWHAs, there is increased utilization of alternative and complimentary medicines in association with HAART, although many of these adjuvants have been implicated in drug-induced liver/other organs’ injury [32]. AP alone at both doses showed a tendency to cause appreciable increase in values of liver enzymes compared with HAART and vitamins C and E. This may contribute to perturbations in liver metabolism in addition to HAART which ultimately may predispose to worsened outcomes, unfortunately. We did observe that ALP levels were mostly reduced in our study (even in HAART alone groups). Discordant outcomes have been seen also in the work of Kayode et al. [33]. Again, elevations in these enzymes is a sensitive signal for liver injury but may not be specific or even relevant clinically as most of these elevations does improve with duration [30].

Evidence that patients with high levels of AST or ALT had a five-fold greater risk of fibrosis or cirrhosis than in patients with lower enzyme levels still remains an unresolved issue. Studies investigating the link between antiretroviral therapy, HIV and hepatitis C co-infection [34] showed no links with hepatic fibrosis in persons treated with ART. Histopathological assessments revealed that in HAART-treated groups as well as HAART with vitamins C and E, there were extensive network of fibrous connective tissue which dominates the disorganized architecture. Moderate fibrotic strands were also observed following adjuvant treatment with AP. Therefore, although the argument that ART causes chronic histological liver disease, we support that long term liver enzyme elevation in addition to other positive indices tilts the balance towards liver fibrosis as supported by Qurishi et al. [35]. These perturbations are unlikely to be mitigated by AP or concomitant treatment with vitamins C and E.

Lipid profile assessment (composite of total cholesterol—TC, triglycerides—TG, and lipoproteins (high HDL) and (low LDL) play critical role in enabling evaluation of cardiovascular outcomes following ART. AP appeared to show diverse actions on lipoproteins with adjuvant use with HAART promoting HDL and lowering LDL and TGs but with no overall effect on cholesterol. On the contrary, AP (higher dose) seemed to execute the reverse effects on the above parameters. These discordances remains to be further clarified but may not be unconnected with the possibility that herb–drug interactions in the former could favor a better outcome whereas a single herb administration is actually perilous on these lipid profile markers in this protocol. We correlated these observations with the PAS-staining intensities of liver sections and there is supporting link. Groups H (AP_200mg/kg_), E (HAART + vitamin E), B (HAART + AP_100mg/kg_) and C (HAART +AP_200mg/kg_) showed the least PAS-staining intensity possibly indicating low deposits of glycogen. These derangements in lipid and glucose metabolism are supported by previous studies (RCT [37]) involving non-NRTIs in regimen but may also be due to other contributory factors like gender and age among others [37].

It is interesting to note that while AP extracts upregulated the levels of LDL and TG, these parameters were lowered when AP was used as adjuvant to HAART. Also, adjuvant use of vitamins C and E upregulated HDL and lowered TG with no significant change in total cholesterol. These opposite effects on lipid profiles alludes to H&E stained sections of the liver where vitamin E showed good cyto-protective effects and adjuvant use of vitamins C and E with HAART was seen to exacerbate hepatic cellular structural injury. These observations agrees with our previous work [23] and are suggestive that the pharmacokinetic interactions between ART and plant-based extracts can adversely alter lipid/lipoprotein levels and may augment the risk for cardiometabolic syndrome.

Whether there is possibility of antiretroviral sequestration/or boosting in hepatic tissues, the exact mechanistic pathway for the negative indices triggered by AP in our study is still a subject for further interrogation. However, the pathogenic pathway for HAART-mediated injury has been associated with mitochondrial toxicity especially with NRTIs [36], [37]. Inhibition of gamma-polymerase is linked with damage to mitochondrial DNA which affects all other organs including the liver [37]. We did not report changes in intracellular glutathione reserve (a major antioxidant defense that protects against toxic insults), it is reasonable to believe that a perturbed antioxidant system could perhaps be a contributory arm towards the inability of AP to mitigate the ravages of HAART in our study.

## 5 Conclusions

While HAART continues to be fundamental in the management of HIV/AIDS in sub-Saharan Africa, the complex issues of toxicities, resistance as well as herbal-drug interactions would continue to be hindrance to achieving the desirable goals. It has emerged from this study that hepatic injuries and perturbations emanating from HAART were not mitigated by AP adjuvants both seen in the histopathological and biochemical assessments. There is no merit in concomitant use of vitamins C and E with HAART also based on the negative outcomes. Therefore, caution is warranted following the over-the-counter use of AP extracts by PLWHAs as immune booster as possibility of exacerbation of hepatic injuries are high. More studies is warranted to further elucidate these findings.

## Conflict of interest

All authors declare that there is no potential conflict of interest.

## Author’s contribution

AOO is the PI and team leader who conceived and designed project, approved final manuscript and lead author; JAI is a PhD student under leader who saw to data collection, analyses and write up; OU is a Masters student under leader who contributed in data collection and analysis; OIO is a PhD student who took part in data analyses, and write up of manuscript; KS is a masters student under team leader who took part in data collection and data analyses and NECS was responsible for interpretation of results, manuscript write up and approval.
